# Patient and family activated escalation systems: a systematic review

**DOI:** 10.1093/intqhc/mzag093

**Published:** 2026-07-08

**Authors:** Noémie Déom, John Welch, Cecilia Vindrola-Padros

**Affiliations:** University College London, London, United Kingdom; NIHR Central London Patient Safety Research Collaboration, London, United Kingdom; NIHR Central London Patient Safety Research Collaboration, London, United Kingdom; University College London Hospitals, London, United Kingdom; University College London, London, United Kingdom; NIHR Central London Patient Safety Research Collaboration, London, United Kingdom

## Abstract

**Background:**

Patient and family activated escalation systems (PFAES) enable patients and families to escalate concerns about in-hospital deterioration and trigger urgent clinical review when standard escalation routes fail. Given limitations of prior reviews and the rollout of Martha’s Rule in England, this systematic review synthesized evidence on PFAES up to 2025, including system types, implementation, stakeholder experiences, sustainability, and equity.

**Methods:**

The protocol was registered on PROSPERO (CRD420250651441) and reporting followed Preferred Reporting Items for Systematic reviews and Meta-Analyses. The databases MEDLINE, Embase, Scopus, CINAHL, and Web of Science were searched (June 2025), with expert input and backward citation searching. Screening was undertaken in Rayyan. Studies reporting empirical data on hospital-based PFAES were included and appraised for quality using the Mixed-Methods Appraisal Tool. Findings were synthesized narratively.

**Results:**

Searches initially identified 6129 papers. Thirty-five studies met the inclusion criteria. PFAES clustered into two activation routes: direct-to-response team models and proxy activation embedded in ward routines. Low awareness (16/35) and limited understanding (15/35) among patients and families were frequently reported and were linked to limited visibility, reliance on written materials, and inconsistent staff explanations under workload pressure. Patients and families commonly described anxiety about speaking up and fear of harming relationships with staff, alongside a preference for healthcare professionals-led escalation due to concerns about bypassing them, viewing activations as outside their patient role, or simply trusting their care team to perceive and escalate deteriorations. Sustainability was associated with governance support, champions, and ongoing promotion, while equity was inconsistently addressed despite language and communication barriers being recurrent.

**Conclusions:**

While only published studies were included in this review, its broader scope and up-to-date coverage identified additional studies and defined two activation models. Direct-to-response team escalation can be a safety net to interprofessional communication failures but often feels too risky or confrontational for patients and families to use. Proxy escalation appears more acceptable yet depends on traditional staff escalation systems, for which direct-to-response team PFAES models are a safeguard. Future research should examine stakeholder perceptions where both routes operate concurrently, such as Martha’s Rule, and determine the organizational conditions that support effective implementation. Implementations should embed equity and cultural safety to ensure vulnerable groups can access and use PFAES.

## Introduction

Patient and family escalation systems (PFAES) enable patients and families to raise concerns about potential or actual deterioration through designated escalation pathways. Most potentially avoidable in-hospital deaths are preceded by abnormal clinical signs, yet timely recognition, escalation, and response to such abnormalities is not reliably enacted by staff [1]. Where standard escalation routes fail, PFAES provide an additional safety net. These systems often emerged following family advocacy after avoidable in-hospital deaths, such as that of Lewis Blackman in the United States, which led to the Lewis Blackman Act [2], and of Ryan Saunders in Australia, which prompted Ryan’s Rule [3], and have since been adopted across paediatric and adult hospital services internationally.

Existing systematic reviews have appraised PFAES but have key limitations. Early reviews drew on a small developing evidence base [4–6]. Later reviews narrowed scope to adult wards [7], paediatric settings [8], or randomized controlled trials [9]. Consequently, no synthesis has yet captured the full breadth of PFAES across all settings, populations, and implementation stages, nor adequately addressed questions of sustainability and equity. The evidence has grown substantially since these earlier reviews, so a comprehensive, up-to-date review is therefore needed. This need is made particularly urgent with the ongoing rollout of a PFAES into national policy. Indeed, Martha’s Rule is implemented at the time of writing across 220 NHS acute sites in England. It introduces a structured daily patient check-in and an always-available route for patients and families in both adult and paediatric settings to request a review by a different team when deterioration concerns are not addressed [10].

To inform this implementation, this systematic review aims to (i) map PFAES globally up to 2025; (ii) examine implementation barriers and facilitators; (iii) explore perceptions and responses among healthcare professionals, patients, and families; (iv) identify operational challenges affecting sustainability and effectiveness; and (v) describe how equity is addressed in PFAES design and implementation.

## Materials and methods

This review was conducted and is reported in accordance with the Preferred Reporting Items for Systematic Reviews and Meta-Analyses (PRISMA) [11, 12]. A review protocol was registered on PROSPERO (Public Registry for Systematic Review Protocols in Health and Social Care) in February 2025 (Registration number: CRD420250651441).

### Search strategy

The search strategy was developed in collaboration with a University College London librarian, refined iteratively, and finalized in June 2025 to capture all potential forms of PFAES. Searches were then cross-checked and expanded using search terms from previously published systematic reviews [4–9]. The final strategy was run in June 2025 in MEDLINE (Ovid), Embase (Ovid), Scopus (Elsevier), CINAHL (EBSCO), and Web of Science (Clarivate), without restrictions to return a global view of existing mechanisms (cf. Supplementary Appendix 1).

### Study selection

#### Eligibility

Studies were included if they provided empirical data on mechanisms allowing patients, family, or carers to escalate concerns about their health in hospital. Excluded articles focused on general patient safety reporting systems without a patient or family-initiated escalation component. Studies conducted in non-hospital settings or lacking empirical data were excluded.

#### Screening

Database search returns were exported to Rayyan, a web-based systematic review management tool [13]. After de-duplication, N.D. screened titles and abstracts against predefined inclusion criteria and documented reasons for exclusion. C.V. checked 25% of excluded papers to verify consistent application of criteria and quality-assure exclusion decisions. Papers that appeared eligible or where eligibility was unclear progressed to full-text assessment. A data extraction form aligned with the review objectives was piloted on five papers to confirm applicability and usefulness, then used for all included papers (Supplementary Appendix 2). Full-text screening, data extraction, and quality assessment were conducted by one reviewer (N.D.). A second reviewer (C.V.) was consulted in cases of uncertainty to minimize the risk of error. A standardized data extraction form was used to minimize subjectivity, and the PRISMA checklist was followed rigorously.

#### Quality assessment

The Mixed-Methods Appraisal Tool (MMAT) was used to assess papers’ quality across a range of study designs, including qualitative, descriptive quantitative, and mixed-methods [14, 15]. The rating of articles is based on five criteria; articles can be scored from 0 to 5, with 5 the highest score, denoting that all quality criteria are addressed.

### Data synthesis

We used narrative synthesis to describe and analyse findings across included papers. Following guidance [16], an extraction form supported initial data familiarization. We then populated objective-specific evidence tables to enable cross-comparison. Vote counting was used to summarize the frequency of barriers and facilitators. These steps supported systematic within- and across-study comparison, and informed narrative themes on barriers, facilitators, perceptions, responses, effectiveness, sustainability, and equity.

## Results

**Figure 1:**
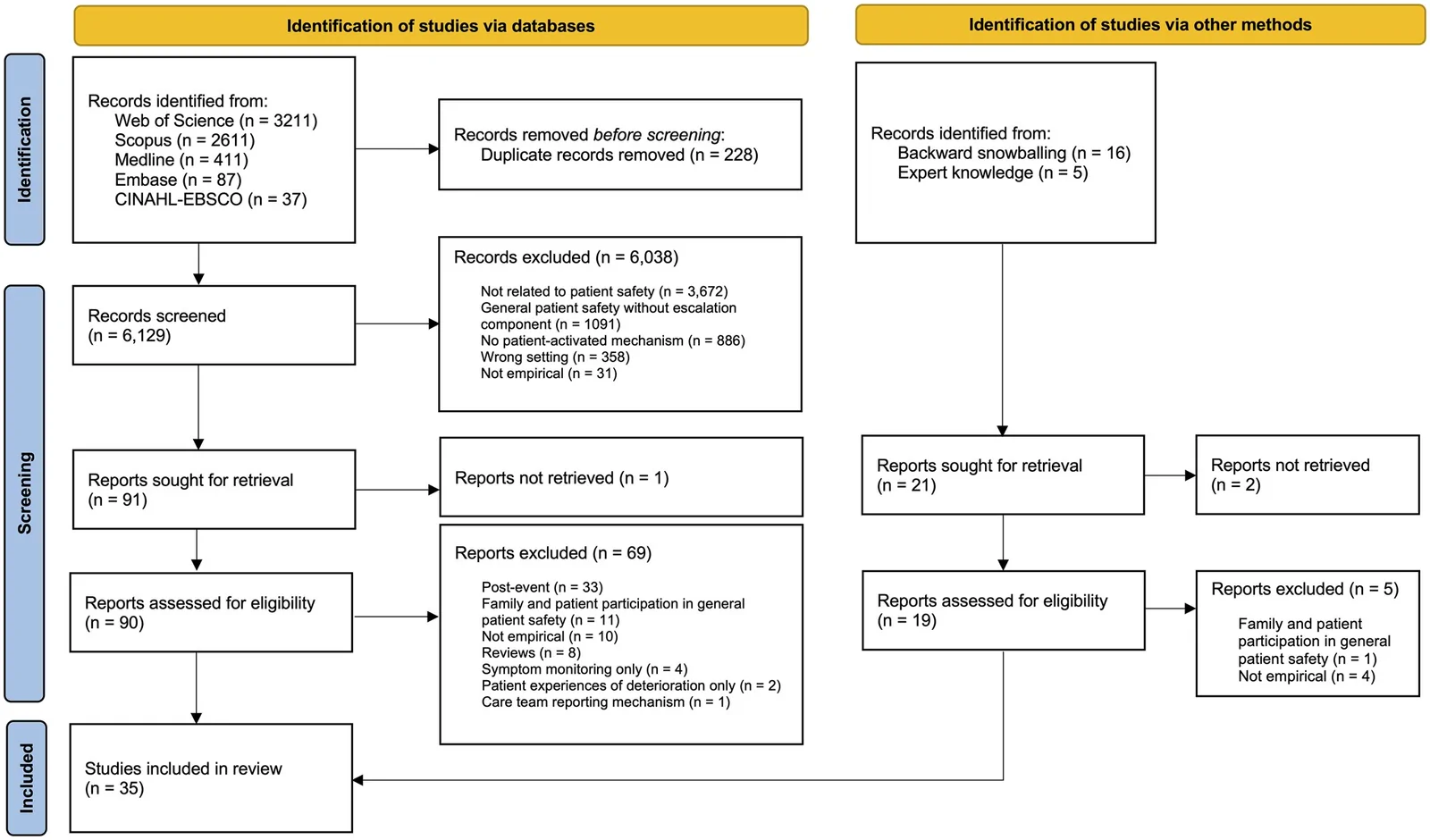
PRISMA flow diagram of study selection and screening.

Database searches identified 6129 papers. After reviewing the full text of 109 papers, 35 studies met the inclusion criteria [17–51] (Fig. 1).

### Characteristics of included studies

Table 1 provides a summary of the characteristics of included studies.

**Table 1:** Characteristics of included studies.

Study (year)	Country	Aim	Method	Care setting	References
Albutt *et al*. (2020)	United Kingdom, England	To develop and test a patient wellness questionnaire to help hospital patients report early signs of deterioration (pre-implementation)	Mixed-methods study	Adult	[19]
Baird *et al*. (2011)	United States, Michigan	To report on the creation, structure, rollout and early outcome of a patient/family-activated concern system	Quantitative study	Paediatrics and adult	[20]
Bavare *et al*. (2018)	United States, Texas	To describe the characteristics, reasons for activation, and outcomes of Family-Initiated Rapid Response (FIRR) events	Quantitative study	Paediatrics	[21]
Bogert *et al*. (2010)	United States, California	To describe how Condition H (family-activated rapid response) was planned, piloted, and rolled out	Quantitative study	Adult	[22]
Brady *et al*. (2014)	United States, Ohio	To report on a QI project to design and test a family-activated MET and assess impact on call volume, ICU transfers, and reasons for activation	Quantitative study	Paediatrics	[40]
Carter *et al*. (2022)	United Kingdom, England	To evaluate clinical utility and acceptability of an electronic paediatric early warning system with embedded sepsis screening (DETECT e-PEWS)	Mixed-methods study	Paediatrics	[23]
Cornell and Datson (2023)	United Kingdom, England	To track the use of ‘Call 4 Concern’, how staff responded, effects on workload and safety, and user feedback over the first year	Mixed-methods study	Adult	[24]
Dwyer *et al*. (2020)	Australia, Queensland	To assess clinicians’ and activators’ experiences to develop an understanding of the incidence, contributing factors, and outcomes surrounding Ryan’s Rule activations	Quantitative study	Paediatrics and adult	[46]
Earle *et al*. (2018)	New Zealand	To report on the piloting and co-designing of a patient/family escalation mechanism (Korerō Mai)	Qualitative study	Paediatrics	[41]
Eden *et al*. (2017)	United States, Pennsylvania	To describe the characteristics of patients activating Condition Help, the reasons for calls, whether changes in care were implemented, and outcomes	Quantitative study	Adult	[32]
Flynn *et al*. (2022)	Australia, Queensland	To evaluate Ryan’s Rule calls in surgical care, looking at why patients/families escalated, what actions followed, and outcomes	Quantitative study	Paediatrics and adult	[39]
Gerdik *et al*. (2010)	United States, Florida	To report the rollout and outcomes of a family and patient activated Rapid Response Team in an adult hospital	Quantitative study	Adult	[18]
Gill *et al*. (2022)	Australia, Western Australia	To design a uniform paediatric system to spot deterioration early and trigger the right response across hospitals	Qualitative study	Paediatrics	[47]
Gill *et al*. (2022)	Australia, Western Australia	To explore the perspectives of family members of Aboriginal children about their involvement in recognizing clinical deterioration in a hospital setting and the effectiveness of a poster designed to promote family involvement	Qualitative study	Paediatrics	[25]
Gill *et al*. (2018)	Australia, Western Australia	To identify barriers and facilitators to implementing a parent escalation of care process	Qualitative study	Paediatrics	[35]
Gill *et al*. (2019)	Australia, Western Australia	To evaluate the implementation of a parent escalation process and checking awareness, use, and views from parents and nurses	Mixed-methods study	Paediatrics	[36]
Guinane *et al*. (2018)	Australia, Victoria	To investigate the experiences of patients who received a medical emergency team review following a period of clinical deterioration and their views about the potential use of a PFAES (pre-implementation)	Qualitative study	Adult	[31]
Heath *et al*. (2016)	United Kingdom, England	To develop and pilot a communications bundle to help parents raise concerns and escalate care for deteriorating children in hospital	Qualitative study	Paediatrics	[26]
Hueckel *et al*. (2012)	United States, North Carolina	To implement scripted family teaching on Condition H and check families’ understanding	Mixed-methods study	Paediatrics	[27]
King *et al*. (2019)	Australia, South Australia	To explore patient and family experiences of reporting deterioration and their views on education for a PFAES	Qualitative study	Adult	[37]
King *et al*. (2014)	Australia, South Australia	To understand clinicians’ views on consumer reporting of patient deterioration through an established hospital PFAES	Qualitative study	Paediatrics and adult	[28]
Marufu *et al*. (2025)	United Kingdom, England	To co-design a multilingual digital app to let parents directly escalate concerns about a deteriorating child to the rapid response team (pre-implementation)	Qualitative study	Paediatrics	[34]
McCawley *et al*. (2013)	United States, North Carolina	To revise Condition H education for staff, patients, and families, and to see if understanding and safety outcomes changed	Quantitative study	Adult	[29]
New *et al*. (2019)	Canada	To explore patient safety from the perspectives and experiences of patients, and to describe willingness to report incidents utilizing an existing safety reporting system	Qualitative study	Adult	[43]
Ngoya Ntumba *et al*. (2023)	United Kingdom, Wales	To map the PFAES evidence, to capture patient views for barriers/opportunities recognition, and to propose a measurement framework	Qualitative study	Adult	[38]
O’Halloran *et al*. (2023)	United States (nationwide)	To describe systems to detect/respond to deteriorating hospitalized children at Pediatric Resuscitation Quality Collaborative (pediRES‐Q) institutions	Mixed-methods study	Paediatrics	[17]
Odell (2019)	United Kingdom, England	To review seven years of patient and family-activated critical care outreach based on referral patterns, reasons for calls, and service feasibility	Quantitative study	Adult	[49]
Odell (2010)	United Kingdom, England	To evaluate a system that allows patients and relatives to access the CCO team directly	Qualitative study	Adult	[30]
Price *et al*. (2018)	New Zealand	To describe the co-design, pilot testing, and implementation of a patient, family, and whānau escalation system	Qualitative study	Paediatrics and adult	[42]
See *et al*. (2014)	Singapore	To test a brief education intervention (AWARE) to help hospitalized adults recognize deterioration and speak up early for patient-led escalation	Quantitative study	Adult	[51]
Strickland *et al*. (2019)	New Zealand	To explore adult patients’ and families’ experiences of deterioration, whether they want a patient and/or family activated escalation service, and what might stop them using it (pre-implementation)	Qualitative study	Adult	[48]
Sutton *et al*. (2024)	United Kingdom, England	To report on the facilitators and barriers to the implementation of a PFAES	Qualitative study	Adult	[33]
Thiele *et al*. (2022)	Australia, South Australia	To explore clinicians’ understanding and perceptions of consumer escalation	Qualitative study	Adult	[44]
Thiele *et al*. (2025)	Australia, South Australia	To explore patient and family knowledge of acute clinical deterioration, and their confidence and perceived barriers to escalating their concerns	Qualitative study	Adult	[45]
Van Voorhis and Willis (2009)	United States, North Carolina	To describe implementation and outcomes of a hospital-wide paediatric early response system (PERT), including addition of family activation	Quantitative study	Paediatrics	[50]

### Quality assessment

The MMAT quality assessment indicated that all included studies had clear research questions with enough data to address those questions. When quality fell, it was due to missing or weak reporting of key methodological details. Mixed-methods studies (*n* = 5) had a lower mean score of 4/5 (range 1–5), largely due to one study providing insufficient details on sampling strategy and inclusivity [24]. Qualitative studies (*n* = 17) scored highly overall (mean 4.64/5, range 2–5), although two studies had a lower score because some of the findings were not clearly evidenced [25, 30]. Quantitative studies (*n* = 13) had a mean score of 3.92/5 (range 1–5), due to incomplete reporting on the risk of non-responses in three papers [22, 27, 50] (Supplementary Appendix 3).

### Synthesis of findings

#### Typology of PFAES

Twenty-two unique PFAES were identified across 35 studies. Ten were from the United States, five from Australia, five from the UK, and the remainder elsewhere (Table 2). The earliest systems emerged in the United States in the early 2000s, with UK and Australian initiatives from 2009 and 2013 respectively.

**Table 2:** Existing PFAES identified in the included studies.

Characteristics					Core design features					Population and setting			References
Name	Country (region/state if stated)	Year of implementation (if stated)	Health system level	Implementation stage	Who can activate escalation	PFAES activation channel	Target responder	Scope	Gatekeeping	Hospital type	Service	Target population	
4-CARE	United States, Florida	2007	Local	Post-implementation (2 years)	Patients (adult); Families	Phone	RRT	Deterioration	Direct	Tertiary teaching hospital	All	Adult	[18]
AWARE	Singapore	2012	Local	Pilot	HCP	In-person	HCP	Deterioration	Primary healthcare team	Acute tertiary hospital	Two general medical-surgical wards	Adult	[51]
Call 4 Concern	United Kingdom, England	2009	Local	Pilot and post-implementation (7 years)	Patients (adult); Families	Phone	CCOT	Escalation of concerns	Direct	District General Hospitals	All	Adult and paediatrics	[24, 30, 49]
Calling 4 Help (C4H)	Australia, Western Australia	2015	Local	Implementation	Families	Phone	MET	Deterioration	Direct	Tertiary paediatric hospital	All	Paediatrics	[35, 36]
Consumer Escalation System (CES)	Australia, South Australia	2019	Local (from national requirements)	Implementation	HCP	In-person	RRT	Escalation of concerns	Primary healthcare team	Public teaching hospital	Medical, surgical, and speciality wards	Adult	[44, 45]
Condition Concern	United States, Michigan	2009	Local	Implementation	Patients; families	Phone	Administrative Associate Manager (forwards to RRT)	Escalation of concerns	Triage	Three not-for-profit major hospitals	Not reported	Adult and paediatrics	[20]
Condition H	United States, California	2007	Local	Pilot	Patients (adult); families; visitors	Phone	RRT	Deterioration	Direct	Tertiary hospitals	All	Adult	[22]
	United States, North Carolina	2007											[29]
Condition H(elp)	United States, North Carolina	2011	Local	Post-implementation (1 year)	Families	Phone	RRT	Escalation of concerns	Direct	Tertiary paediatric hospital	Two inpatient paediatric units	Paediatrics	[27]
Condition Help (CH)	United States, Pennsylvania	2005	Local	Post implementation (3 years)	Patients (adult); families	Phone	RRT	Escalation of concerns	Direct	Tertiary teaching hospital	All	Adult and paediatrics	[32]
Consumer-initiated escalation-of-care (CIEoC)	Australia, South Australia	2019	Local	Post-implementation (3 years)	Patients; families; visitors	Phone	RRT/MET	Deterioration	Direct	Two hospitals (Trauma Medical Centre; acute Care Hospital)	All	Adult and paediatrics	[28]
Detect E-PEWS	United Kingdom, England	2020	Local	Implementation	HCP	Digital (app)	Ward clinical team and RRT	Deterioration	Primary healthcare team	Tertiary paediatric hospital	All	Paediatrics	[23]
Early 3S’ (RESPOND)	United Kingdom, England	2022	Local	Implementation	Patients (adult)	Digital (buzzer)	CCOT/MET	Escalation of concerns	Direct	Teaching hospital, rural DGH, urban DGH	Selected wards (4 total)	Adult	[33]
ESCALATION	Australia, Western Australia	2021	Regional	Implementation	HCP	In-person	Case-by-case, based on EWS	Deterioration	Primary healthcare team	Tertiary paediatric hospital	All	Paediatrics	[25, 47]
Family Activated MET	United States, Ohio	2007	Local	Implementation; post-implementation (6 years)	Families	Phone	MET	Escalation of concerns	Direct	Tertiary paediatric hospital	All non-ICU in-patient units	Paediatrics	[40]
Family-Initiated Rapid Response (FIRR)	United States, Texas	2009	Local	Post-implementation (3 years)	Families	Phone	RRT	Escalation of concerns	Direct	Tertiary paediatric hospital	Non-critical care inpatient units	Paediatrics	[21]
Korero Mai	New Zealand, Canterbury	2018	National	Implementation	HCP	In-person	Primary HCP	Escalation of concerns	Primary healthcare team	Tertiary regional hospital	Paediatric progressive care/high dependency unit	Paediatrics	[41]
	New Zealand, Waitemata	2018			Patients; family; whānau	Phone and in-person	Independent clinical reviewer and Primary HCP		Direct and primary healthcare team	District Health Board	Not reported	Adult and paediatrics	[42]
Listening to You	United Kingdom, England	2014	Local	Implementation	HCP	In-person	Ward clinical team and PACE	Deterioration, communication bundle	Primary healthcare team	Tertiary paediatric hospital	Four inpatient wards	Paediatrics	[26]
PARR	United Kingdom, Wales	2023	Local	Implementation	Patients (adult); Families	Phone	RRT	Escalation of concerns	Direct	District General Hospital	Single ward and discharged patients	Adult	[38]
Pediatric Care BefOre Deterioration Events (CODE)	United States	Not reported (data collection started 2021)	National	Not reported	HCP	In-person	Primary HCP	Escalation of concerns	Primary healthcare team	30 medical centres (including 21 paediatric hospitals)	Paediatrics	Paediatrics	[17]
Pediatric rapid response system (PRRS)	United States, North Carolina	2005	Local	Implementation	Families	Phone	RRT	Deterioration	Direct	Tertiary paediatric hospital	All	Paediatrics	[50]
Ryan’s Rule	Australia, Queensland	2013	Regional	Post-implementation (5 years)	Patients; Families; Friends	Phone	Government hotline > hospital contact (forwards to clinical review)	Deterioration	Triage	Ten hospitals	Surgical ward	Adult and paediatrics	[39]
				Post-implementation (2 years)	Patients; families	Phone	Independent clinical reviewer		Direct	Tertiary regional hospital	All		[46]
Stop-alert; assess-fix-eliminate; escalate and report	Canada, Saskatoon	Not reported (data collection started 2017)	Local	Post-implementation (missing year of implementation)	Patients (adult); Families	Phone	Primary HCP	Escalation of concerns	Direct	Urban tertiary care centre	One surgical and three medical inpatient wards	Adult	[43]

Five studies described PFAES at pre-implementation stages (5/35, 14%) (Table 3). Among implemented PFAES (30/35, 86%), most were described during implementation or within 1 year post-implementation (20/30, 67%). Ten were reported 2–7 years post-implementation (10/30, 33%). Where duration was reported (9/10, 90%), the mean time since implementation was 3.5 years.

**Table 3:** PFAES in pre-implementation, identified in the included studies.

Study characteristics					Description	References
Country (region/state if stated)	Year of publication (if stated)	Hospital type	Service	Target population		
United Kingdom, England	2020	Teaching Hospital	Three high cardiac-arrest wards	Adult	Mixed-methods pre-implementation study. Development and pilot of a Patient Wellness Questionnaire for inpatients to rate their wellness during routine observations, adding a patient-reported cue for possible deterioration and escalation.	[19]
Australia, Victoria	2017	Two hospitals (one private and one public)	Medical and surgical units	Adult	Qualitative pre-implementation study. Exploration of adult inpatients’ experiences of MET review and their views on their potential role in escalation of care.	[31]
New Zealand, Auckland	2018	Tertiary metropolitan hospital	Two wards: medical and surgical	Adult	Qualitative pre-implementation study. Exploration of patients’ and families’ experiences of acute deterioration, their perceived need for a patient and/or family-activated escalation service, and potential barriers to activating such a service.	[48]
United Kingdom, England	2024	Tertiary Paediatric Hospital	All inpatient units	Paediatric	Qualitative co-design pre-implementation study. Development of a multilingual app-based paediatric FARR system for parents/carers to escalate deterioration concerns directly to the paediatric critical care outreach team.	[34]
Australia, South Australia	2019	Acute hospitals in South Australia	All inpatient units	Adult	Qualitative pre-implementation study. Exploration of patient and family experiences of reporting deterioration and their views on education and preferred models for a consumer reporting mechanism.	[37]

Systems clustered into two activation routes: direct-to-response team and proxy activation. Direct-to-response team models allow patients and families to access an external response team. They bypass the primary team, although the external team will usually liaise with the primary team. Proxy activation models embed escalation within bedside routines and early warning score (EWS) processes. Patients and families raise concerns to ward staff who should escalate on their behalf. They differ from standard staff escalation routes because they are supported by explicit policies giving patients and families the right to trigger escalation.

Across paediatric services, parents and carers were consistently positioned as the main activators, alongside staff. No study explicitly described children or young people themselves as direct activators. In adult services, patients were more often named as potential activators, followed by family members. Some policies also include other visitors. In all studies, escalation mechanisms are exclusively triggered by adults.

#### Barriers and enablers to the implementation and use of PFAES

##### Reach and comprehension

Sixteen studies (45%, 16/35) reported low patient and family awareness of PFAES. This was linked to limited visibility, unclear messaging, and reliance on written materials. Health care professionals’ (HCPs) explanations were inconsistent because of time pressures, patients being too unwell, or relatives being absent. Three studies described staff selecting who received information based on perceived levels of families’ and patients’ stress, whether the patients appeared well, or whether the patients or families were judged likely to use the process inappropriately.

Fifteen studies (43%, 15/35) reported limited understanding of PFAES. Families and patients were unsure when escalation was warranted and who to contact. Two studies reported that face-to-face explanations improved understanding. Enablers included clear staff explanations and repeated, multimodal prompts supported by materials that families and patients could review (Tables 4 and 5).

**Table 4:** Reported challenges and enablers to the comprehension and reach of PFAES.

Domain	Finding	Detail	Studies (*n*)	References
**Comprehension and reach of PFAES: *Challenges***				
Low awareness	Posters and leaflets	Limited visibility or unclear messaging	6	[22, 24, 26, 42, 43, 45]
		Only source of patient/family information	4	[27, 33, 45, 50]
	Inconsistent HCP communication	Patient too unwell to receive information	10	[19, 24, 28, 33, 38, 42, 43, 45, 49, 51]
		Time constraints	6	[28, 33, 35, 36, 40, 42]
		Family member not present	2	[36, 50]
		Selective informing based on perceived wellness, stress, or risk of inappropriate use	3	[35, 36, 44]
Limited understanding	Poor education	Inadequate explanation of activation process	9	[22, 23, 26, 31, 39, 40, 42, 43, 51]
		Uncertainty about when to escalate and who to contact	6	[26, 29, 33, 42, 43, 51]
		Leaflets too wordy or overwhelming, particularly at admission	4	[25, 33, 37, 41]
		HCPs filter information at admission to avoid overburdening families	2	[35, 36]
HCP education challenges	Workload pressures	HCPs forget or choose not to provide PFAES information	7	[27, 28, 33, 35, 36, 40, 42]
		Education time-consuming and difficult to prioritize	5	[27, 28, 33, 36, 40]
		Patients and families feel less listened to	3	[19, 36, 39]
	Unclear role ownership	Limits consistent PFAES education delivery across professional roles	3	[28, 35, 42]
Low health literacy	Differential access and confidence	PFAES may be used more readily by more educated and confident families	5	[28, 33, 36–38]
		Limited health literacy discourages questioning of clinicians	4	[35, 36, 39, 45]
		Difficulty recognizing deterioration, judging severity, translating symptoms into actionable information	2	[25, 38]
**Comprehension and reach of PFAES: *Enablers***				
Multimodal education	Delivery strategies	Diverse locations combining written and visual materials	5	[21, 22, 25, 26, 34]
		Face-to-face HCP-delivered explanations	5	[22, 34, 36, 38, 48]
		Repeated visual prompts in the care environment	7	[21, 36, 37, 40, 42, 48, 49]
		Conversational explanations from staff preferred	6	[22, 34, 35, 37, 48, 50]
		Education timed to points of greatest receptiveness	2	[29, 37]
		Verbal explanations reinforced by written materials families could revisit	4	[22, 34, 41, 48]
Co-design	Implementation with patients, families, and HCPs	*Not specified*	8	[25, 26, 33, 34, 37, 40, 41, 47]
		Iterative prototype review of posters and flyers, including plain-language and naming refinement	4	[25, 26, 34, 47]
		Mapping education and activation pathways	2	[40, 41]
		Testing resources with culturally and linguistically diverse consumers	2	[25, 37]
		Improved acceptability of materials	2	[25, 42]
**Relational and hierarchical dynamics: *Challenges***				
Communication with HCPs	Power differentials	Insufficiently relational/responsive communication discourages questions	6	[19, 22, 23, 26, 34, 39]
		Activation dependent on confidence to challenge traditional hierarchies	6	[35, 36, 42, 43, 45, 48]
	Patient reluctance	Patients/families feel vulnerable and less able to question clinical opinions	5	[31, 42, 44, 45, 48]
		Patients unwilling to override clinicians; do not view activation as their role	1	[31]
Interprofessional communication	Staff escalation failures	HCPs concerned about being judged for escalating on behalf of patients/families	4	[34, 42, 43, 47]
		Delays and problems in clinical escalation preceding patient/family activation	3	[36, 39, 41]
**Relational and hierarchical dynamics: *Enablers***				
PFAES supports communication	Relational benefits	Formalizes recognition of patient and family knowledge and observations	10	[19, 23, 26, 28, 34–36, 41, 44, 47]
		Improved communication between patients/families and HCPs	9	[24–26, 36, 37, 42, 44, 46, 47]
		Earlier attention to concerns	7	[19, 26, 38, 41, 44, 47, 48]
		Validates concerns and provides reassurance	7	[26, 30, 31, 35, 38, 45, 48]
		Addresses aspects of safety culture	3	[34, 44, 48]
**Organization-level: *Challenges***				
Systemic barriers	Implementation difficulties	Making a whole-system organizational change	3	[35, 42, 47]
		Designing a system that works across specialties, services, and existing escalation pathways	2	[23, 47]
		Difficulties maintaining HCP engagement over time	1	[49]
**Organization-level: *Enablers***				
Governance and infrastructure	Implementation supports	Iterative piloting, audit, and feedback to evaluate fit and refine implementation	13	[21, 22, 24, 27, 29, 33, 39–41, 46, 47, 49, 50]
		Co-design approaches	8	[25, 26, 33, 37, 40–42, 47]
		PFAES usefulness justified to HCPs through evidence and adverse event narratives	5	[18, 22, 37, 41, 49]
		Integration facilitated by existing safety infrastructure	4	[19–22]

**Table 5:** Multimodal material education strategies mentioned in included papers.

Material or channel	Setting	Specific placement	References
Posters	Patient room	Bedside or in-room	[29, 33, 37, 40, 41, 48]
	Ward/shared areas	Nursing stations	[33]
		Waiting rooms	[37]
		Toilets	[37, 42]
	Hospital-wide public areas	Entrance	[49]
		Lifts	[32, 42]
	*Unspecified*	*Unspecified*	[20, 24, 50]
Stickers	Patient room	Phone	[21]
	Staff-facing	On staff	[42]
Phone token (to use phone)	Patient room	*Unspecified*	[21]
Cards	Patient room	*Unspecified*	[42, 48]
Flyers	Unspecified	*Unspecified*	[21]
Resource pack	Patient room	*Unspecified*	[30]
Admission pack	Admission materials	*Unspecified*	[22, 29, 32, 42, 44, 50]
Online information	Online	Hospital website	[34, 42, 49]
		Mobile app	[34, 42]
		*Unspecified*	[44]
RRT staff uniform	Staff visual cue	Uniform	[18]
Bilingual materials	Accessibility	Language adaptation	[29, 42]

##### Equity and capability

Low health literacy was a barrier in nine studies (26%, 9/35), sometimes compounded by language barriers and cultural norms discouraging the questioning of clinicians. Some written materials were too technical or inaccessible. Staff raised concerns that more educated and confident families may use PFAES more readily, supporting calls for plain language, visual, and bilingual resources. Education was time-consuming for HCPs and difficult to prioritize, particularly in high-workload contexts, with unclear role ownership contributing to inconsistent provision. Material and naming were developed and refined using co-design methods, with reported improvements in acceptability and cultural relevance (Table 4).

##### Relational and organization factors

Twelve studies (34%, 12/35) described relational and hierarchical dynamics discouraging activation, including power differentials, perceived low staff responsiveness, and reluctance to override clinicians. HCPs reported concerns about being judged for escalating in proxy models. Three studies described delayed escalation or communication breakdowns preceding direct patient/family activation. Conversely, 10 studies (28%, 10/35) described PFAES as legitimizing patient/family observations and improving communication, reassurance, and earlier attention to concerns. Organizational constraints included sustaining staff engagement and designing systems across pathways. Implementation was facilitated when PFAES were integrated into existing safety infrastructure, supported by piloting, audit feedback, and justified to staff and leaders using adverse-event examples and evidence (Table 4).

#### Perception and responses

##### Healthcare professionals

Twenty studies reported HCPs’ views (20/35, 57%). Before implementation, HCPs expected increased activations and added pressure on already constrained services, affecting rapid response workload and resourcing. Post-implementation, direct PFAES activations were low-volume and a small proportion of rapid response activity. Concerns included challenged ward authority, with worries rapid response teams might overturn ward plans, and that escalation could undermine ward staff judgement and strain patient/family–HCP relationships. Two studies (2/20, 10%) reported nurses’ concerns about professional repercussions if a family or a patient under their care escalated concerns. At ≥1 year, scepticism persisted about the feasibility or value of PFAES during acute deterioration, especially in wards with restricted visiting hours when no visitors could act on the patient’s behalf. Two studies (2/20, 10%) reported biased PFAES communication, including selective informing based on perceived benefit or misuse and descriptions of some families as ‘problematic or demanding’. Some staff reframed ‘concerns’ as ‘complaints’. However, seven studies reported positive views, aligning PFAES with family-centred care. They described PFAES as an added safety layer that could enable faster recognition and responses to deterioration (Supplementary Appendix 4, Table 6a)

##### Patients and their families

Thirty-one studies reported patient and family views (31/35, 89%). In five (5/31, 16%), participants felt escalation enabled rapid responses and improved safety. Supportive messaging provided reassurance. Some wished they had known about the service earlier. Across 16 studies (16/31, 51%), patients and families described anxiety, powerlessness, or frustration about speaking up, linked to lacking knowledge, legitimacy, or language to articulate ‘something is wrong’. They worried about damaging relationships with clinicians, seeming impolite, reprisals after escalation, and burdening busy staff. In four proxy activation studies, patients and families doubted staff would act on their reports. Four studies (4/31, 12%) found patients preferred HCPs to escalate for them, linked to a culture of compliance, or preferring indirect escalation rather than bypassing the primary team. Some trusted HCPs or viewed the service as unnecessary. Others saw activation as outside their knowledge or responsibility. Confidence to speak up was associated with HCP invitation and clear communication about roles and rights (Supplementary Appendix 4, Table 6b).

#### Sustainability

Across post-implementation studies (31/35, 88%), sustainability was linked to formal governance support. Mechanisms included champions and multimodal staff training with updates and implementation guidance. Structured assessment tools and follow-up rounds supported staff ability to respond to family concerns. Normalization and continuous promotion sustained awareness and use.

Consistency weakened when local leadership or champion support was limited or declined, especially where there were no local clinical champions in place, and routine promotion and education relied on already strained ward staff. Staff understanding was fragile when roles were unclear, or implementation efforts were unsustained.

Later studies, although representing only 31% (11/35) of included studies, often reported effectiveness problems, notably delayed or uncertain responses after activation due to pathway failures and slow follow-up. Reliable response depended on capacity and organizational backing, including protected time, dedicated teams, and continued management support.

Earlier studies more often reported operational issues, including interprofessional communication breakdowns and delayed clinician responses. Hierarchies and professional boundaries sometimes created uncertainty about roles and patients and families’ involvement. One study reported nurses using direct escalation routes as workarounds to obtain urgent reviews. In contrast, integrating PFAES ‘prompts’ into routine observations and nursing practices supported ongoing dialogue and normalized patient input, although staff noted time pressures (Supplementary Appendix 4, Table 7).

#### Equity

Equity and access were inconsistently reported. Of 35 studies, seven had diverse participant samples (7/35, 20%), two acknowledged limited data on underserved patients and families (2/35, 5%), three excluded non-English speakers (3/35, 8%), and eight reported demographic data such as gender but not ethnicity (8/35, 22%).

Ten studies reported language barriers limiting access to information for non-English speaking families (10/35, 28%). One study reported staff were less likely to inform these families about PFAES. Proposed improvements included multilingual, culturally adapted, and visually accessible resources, alongside better translation and interpreting services.

Four studies reported differential HCP communication with underserved groups (4/35, 11%). Six studies described patient and family reluctance to question healthcare teams due to sociocultural norms (6/35, 17%). Some patients relied on family or faith-based supports for escalation. Others viewed PFAES as a safety net for relatives rather than something they would use themselves or reported discomfort in hospital settings. Proposed approaches included organizational support for equitable engagement and explicit messaging that legitimized escalation and gave ‘permission’ to speak up. One study called for further research on use and access needs among underserved groups.

Three studies described equity-oriented design or implementation approaches (3/35, 8%). Three initiatives engaged underserved groups during implementation (3/35, 8%). One co-implemented PFAES with Māori, Pacific communities and people with lived experience of disability, improving engagement and staff training. Three studies reported practical adaptations, including interpreter-supported teaching, multilingual and voice-to-text tools (3/35, 8%) (Supplementary Appendix 4, Table 8).

## Discussion

### Key findings

Across 22 unique PFAES, systems clustered into two routes: direct-to-response team (patients/relatives call an external response team) and proxy activation (patients/relatives raise concerns to ward teams, to escalate on their behalf). Patient and family awareness and understanding of PFAES were generally fragile. Studies reported low awareness and limited understanding, due to reliance on written materials that were complex or not visible, inconsistent staff explanations, and staff ‘filtering’ who received information. Multi-modal, repeated, staff-supported education was consistently proposed as a solution. Patients and families were reassured by the option of being able to escalate, but were also anxious about speaking up and feared harming relationships with HCPs. Relational dynamics within HCP-patient’s and interprofessional interactions often discouraged speaking up. HCPs raised concerns about inappropriate use, workload, and challenges to ward authority. Sustainability relied on governance and leadership support, champions, training, routine embedding, and ongoing promotion. Equity was inconsistently addressed, despite known language barriers and differential staff–family interactions, with few studies reporting equity-oriented co-design or adaptations to improve accessibility.

### Interpretation and comparison with the previous literature

This review’s broader scope and up-to-date coverage identified studies not captured in earlier syntheses. The most recent PFAES review highlighted a UK evidence gap [8]. We found seven additional studies, including four from the UK, and identified two activation models: direct-to-response team and proxy activation.

Patients and families often experienced anxiety and disempowerment in direct-to-response team escalation. They reported uncertainty about having the knowledge, legitimacy, or language to voice concerns, and worried about appearing impolite, burdening busy staff, harming relationships with HCPs or triggering reprisals. In comparison, proxy activation appeared more acceptable as it is viewed as less confrontational. This is a significant challenge: direct-to-response team models were introduced to reduce delays caused by hierarchies and poor staff communication [35, 52]; but proxy models can reproduce the vulnerabilities that motivated direct-to-response team designs. By relying on interprofessional escalation of patient and family concerns, they are vulnerable to inadequate handover, variability in staffing, and hierarchical tension between team members, all of which were identified as a leading cause of serious patient harm, accounting for between 52% and 70% of adverse events [53, 54]. Indeed, qualitative research from the UK and United States describes persistent behavioural and systemic inconsistencies in how ward staff recognize and escalate deterioration [55, 56]. Direct-to-response team models were designed specifically to bypass these failures and provide a safety net when intra- and interprofessional communication breaks down. However, the vulnerabilities these models were intended to address remain in place where discomfort, unfamiliarity, or inaccessibility limit patients’ and families’ ability to engage with them.

This review’s findings also add to evidence suggesting that patients’ ability to raise concerns depends heavily on the quality of their relationships with HCPs [57], and that participation in care is affected by staff attitudes, structural barriers, and patients’ own sense that their concerns lack legitimacy or will not be met with empathy [58].

Many studies proposed education as the primary strategy for supporting activation by patients and families, and for encouraging staff to respond to their concerns appropriately. Patients and families reported that face-to-face explanations from HCPs improved both their awareness and understanding of PFAES. Unfortunately, staff frequently lacked the capacity to provide education beyond admission, while admission itself was described as a high-stress period in which information overload reduced patients’ recall and comprehension. Multimodal delivery and repeated education were proposed as solutions, but this places additional demands on a stretched workforce.

Yet, across studies, resource constraints were a recurring theme in patient, family and staff accounts, and in discussions of system sustainability. This is consistent with broader evidence that ward strain is a major patient safety issue requiring structural responses [59]. In this context, education-focused strategies risk increasing the burden they seek to palliate, relying on stretched staff to sustain a mechanism needed because staffing and communication pressures can cause existing escalation processes to fail. This has implications for what education can realistically be expected to deliver, although it remains a practical step towards shifting safety culture and legitimizing patient and family participation. It needs, however, to be embedded within wider organizational change–including protected time and sustainable resourcing—to be effective in practice.

Importantly, families least likely to receive adequate explanation are often those facing the greatest barriers to activation. Several studies reported HCPs’ selective informing of patients and families based on perceived illness or wellness, assumed stress, or concerns about inappropriate use. This may mean that the most overwhelmed or vulnerable families are less likely to receive information. Additionally, patients and families described as more vulnerable also reported reluctance to activate, unwillingness to bypass ward staff, and uncertainty about what levels of concern justified use. In this context, not receiving PFAES information is likely to compound existing barriers to activation. Wider evidence confirms that vulnerable populations face barriers to care that translate into measurably worse outcomes, even where safety systems are in place [60–63]. These findings suggest equity of access should be a core implementation concern, and that inequity in PFAES demands particular attention given that these systems function as a safety net of last resort. Efforts should go beyond producing accessible materials. This includes embedding co-design with underrepresented communities as a design standard rather than an optional exercise, systematic interpreter provision rather than ad hoc arrangements, and staff training specifically aimed at counteracting gatekeeping behaviours. Taken together, these measures would support more equitable reach and help ensure implementation is responsive to the needs of the communities it serves.

### Strengths and limitations

This review used a deliberately broad search to reduce the risk of missing systems that are inconsistently labelled. Including pre-implementation studies strengthened interpretability by capturing early evidence on perceived need and feasibility of PFAES. Limitations remain. Inconsistent definitions and naming may have left information gaps. This review was also limited by single-reviewer data extraction and quality assessment, which may have introduced selection bias. We searched published studies only, so findings are shaped by the academic literature and may under-represent systems described in grey literature or under-report implementation failures. Despite no language restrictions, included studies were from English-speaking countries, limiting transferability to settings with different health system structures and communication norms. Finally, the models proposed in this review inductively arose from included studies and need testing to be verified.

### Implications for research, policy, and practice

Future research should examine how activation works in real-world settings. Policies such as Martha’s Rule, which combine both direct-to-response team and proxy models, offer an opportunity to examine whether routine staff-initiated prompts improve patients’ and families’ perceived legitimacy, confidence to escalate, and actual use. Research is also needed to determine the structural and organizational conditions in which education-focused implementation strategies are effective. Given staff time constraints and patients and families’ difficulties absorbing information at admission, alternative timepoints when education may be easier for HCPs, and more accessible for patients and families should be identified. At policy level, promoting PFAES as public knowledge may improve awareness before hospitalization and reduce dependence on bedside explanations.

Efforts should also focus on tackling wider organizational cultures and working conditions that affect both how staff communicate with each other and how confident patients and families feel about speaking up. Similarly, given that resource constraints were a recurring theme across patient, family, and staff accounts in this review, health economic and workforce research is needed to determine whether, and under what conditions, staffing pressures that motivate the creation of PFAES also undermine their sustained implementation. This is especially relevant to Martha’s Rule as a multi-model patient safety policy.

Equity and cultural safety should be routine design and implementation considerations to create culturally secure hospital environments and accessible escalation systems. This includes interpreted and translated materials in accessible formats, staff training to mitigate bias and gatekeeping, and co-design with a representative sample of the local patient population. Paediatric services require particular attention. Existing evidence describes escalations as solely adult-triggered, indicating a need for approaches that support children and young people’s participation in escalation conversations. Systematic evaluation of equity-focused designs and implementation strategies is needed, with findings disseminated regardless of outcome. This is particularly pressing given the scarcity of such evidence and the risk that without it, vulnerable groups will be excluded by the very system designed to protect them.

## Conclusions

This review identified 22 PFAES across 35 studies, clustering into direct-to-response team and proxy activation models. Across settings, reach and comprehension were fragile, limited by low awareness, reliance on written materials, and inconsistent or selective staff explanations. Patients and families found direct-to-response team escalation uncomfortable. Proxy routes were felt more acceptable but vulnerable to delays or filtering when staff were busy. Where systems were well implemented, patients and families reported feeling reassured, legitimized in raising concerns, and more confident in their care. Staff raised concerns about workload, suitability, and ward authority. Sustainability depended on governance, champions, training, routine embedding, and ongoing promotion. Equity was inconsistently addressed despite recurring language barriers and differential interactions. Implementation should prioritize multimodal, repeated education that legitimize escalation, culturally safe design. Further research should evaluate combined PFAES models in real-world settings, determine the organizational conditions that support implementation, and systematically assess equity-focused designs to ensure vulnerable groups are not left behind by the systems intended to protect them.

## Supplementary Material

mzag093_Supplementary_Data

## Data Availability

We have provided supplementary data files with information regarding studies collected and screened in the Supplementary Material.
