# Supplementary material for: Patient and family activated escalation systems: a systematic review
Source: Int J Qual Health Care. 2026 Jul 8;38(3):mzag093. doi: 10.1093/intqhc/mzag093 (PMC13458593; doi:10.1093/intqhc/mzag093)
Supplement: Supplementary file 1 [file mzag093_supplementary_data.docx]

# Supplementary material

## Appendix 1: Search terms

Searches combined three concept blocks: (1) activators (i.e. *patients*, *families*), (2) escalation mechanisms and known named mechanisms (i.e. *Ryan’s Rule*, *PFAES*, *PARM*, etc.), and (3) keywords drawn from the review’s objectives (i.e. *barrier*, *enabler*, *equity*, *access*). Because no Medical Subject Headings (MeSH) terms exist for patient-activated reporting mechanisms, related subject headings (i.e., *patient participation*, *shared decision making*, *patient safety*, *escalation of care*) were incorporated.

((((activat* OR led OR initiat* OR trigge* OR driven OR trigger OR engag* OR launch* OR instigate* OR prompted OR motivat* OR warn* OR involv* OR voice OR narrative* OR feedback OR perspective OR opinion OR view OR input OR say OR account OR descri* OR input OR collaborat* OR participat*) AND

(carer* OR patient* OR family OR guardian OR "service user" OR "healthcare user" OR inpatient* OR relative* OR kin OR partner* OR custodian* OR self OR wife OR husband OR child* OR friend)) OR

( “Martha’s Rule” OR “Ryan’s Rule” OR “Lewis Blackman” OR “ Call 4 Concern” OR “REACH" OR "Aishwarya" OR "Korero Mai" OR “Kōrero Mai” OR "Talk to me")) AND

(("rapid response" OR escalat* OR alert OR raise* OR communicat* OR speak up OR signal* OR report* OR deterioration OR response OR "medical emergency team" OR "rapid response team" OR “critical care outreach" OR "clinical emergency response" OR "urgent intervention team" OR "early intervention team" OR "resuscitation team" OR MET OR RRT OR CCOT OR PCCOT OR recognition* OR concern* OR worr* OR safety OR "safety net") AND

(ward OR hospital OR "intensive care" OR clinical OR inpatient OR "advanced care" OR "secondary care" OR "tertiary care" OR acute OR "critical care"))) AND

((challeng* OR limit* OR constrain* OR difficult* OR resist* OR block* OR hurdle OR issue OR problem OR negative OR impediment OR reluctan* OR barrier* OR gap OR delay OR breakdown) OR

(enable* OR support* OR aid* OR promot* OR drive* OR contribut* OR incentive* OR resource OR positive OR success* OR facilitat*) OR

(influenc* OR uptake OR integrat* OR embed* OR roll-out OR use OR appl* OR execut* OR deliver* OR operationalis* OR practice OR workflow OR staff* OR delivery OR implement*) OR

(equalit* OR equit* OR justice OR fairness OR inclusiv* OR equit* OR discriminat* OR inequalit* OR inequit* OR imbalance OR disparities OR underserved OR vulnerable OR non-discrimination OR disadvantage* OR minority OR racial* OR low-income OR disabl*))

**Additional MeSH terms used, where available:**

- Disease Progression;
- *Emergency Treatment;
- *Family; Health
- Knowledge, Attitudes, Practice
- Patient Participation [*methods];
- *Patient Safety;
- Symptom Assessment [methods]
- 'involvement in escalation of care'
- 'attitudes and beliefs’
- Shared decision-making (SDM)

These searches were done without date or language restrictions.

## Appendix 2: Methodological details

Full texts were assessed against the inclusion criteria. Where eligibility remained uncertain after full-text review, CV and ND discussed the paper until consensus was reached. Because the initial screening showed that PFAES are described in many ways and not always explicitly, we consulted experts for advice on relevant publication and backward snowballing to the search strategy. This involved checking the reference lists of relevant peer-reviewed systematic reviews and all included full-text papers to identify additional studies and capture all possible iterations of PFAES. Consulting experts and backward snowballing stopped when data saturation was reached.

The following descriptive information was extracted from each articles: publication characteristics (title, name of authors, document type, and year of publication); study characteristics (country of study, study setting, scope or focus, study design, study population, study timeline, comparator group, and sample size); intervention characteristics (PFAES name or type, description of PFAES); PFAES implementation process (description of implementation, barriers, facilitators, healthcare professional perceptions and responses, patient and families perceptions and responses, equity considerations); outcomes and impact (description of effectiveness, impact on patient safety, patient and family satisfaction, description of sustainability).

For included papers, a data extraction form was developed based on the review objectives. The data extraction table was initially piloted on a subset of five papers to ensure its applicability and usefulness. The form was then used to extract data from all included papers.

## Appendix 3: Quality appraisal

**Quality appraisal summary table**

| Study design | n | Mean MMAT score (range) | Most common criteria met | Most common criteria not met or unclear | Studies scoring < 4 (n) |
| --- | --- | --- | --- | --- | --- |
| Qualitative studies | 17 | 4.64/5 (2-5) | Appropriate qualitative approach (1.1); adequate data collection methods (1.2); coherence between data sources and analysis (1.5) | Findings not clearly derived from (1.3) or sufficiently substantiated by data (1.4) | Gill et al., 2022; Odell et al., 2010 (2) |
| Quantitative descriptive studies | 13 | 3.92/5 (1-5) | Appropriate measurements (4.3); adequate statistical analysis (4.5) | Risk of non-response bias (4.4); sampling strategy and representativeness (4.2) | Bogert et al., 2010; Hueckel et al., 2012; Van Voorhis & Willis, 2009 (3) |
| Mixed-methods studies | 5 | 4/5 (3-5) | Adequate rationale for mixed-methods design (5.1); effective integration of components (5.2) | Interpretation of integrated outputs (5.3); divergences between quantitative and qualitative results (5.4); adherence to quality criteria of each tradition (5.5) | Cornell & Datson, 2023 (1) |

**Full quality appraisal table**

|  | **MMAT SCORE /5** | **SCREENING QUESTIONS** | | **1. QUALITATIVE STUDIES** | | | | | **4. QUANTITATIVE DESCRIPTIVE STUDIES** | | | | | **5. MIXED METHODS STUDIES** | | | | |
| --- | --- | --- | --- | --- | --- | --- | --- | --- | --- | --- | --- | --- | --- | --- | --- | --- | --- | --- |
|  |  | S1 | S2 | 1.1. | 1.2. | 1.3. | 1.4. | 1.5. | 4.1. | 4.2. | 4.3. | 4.4. | 4.5. | 5.1. | 5.2. | 5.3. | 5.4. | 5.5. |
| Albutt et al., 2020 | **4** | Y | Y | Y | Y | Y | Y | Y | Y | N | N | N | Y | Y | Y | Y | Y | N |
| Baird and Turbin, 2011 | **4** | Y | Y |  |  |  |  |  | Y | Y | N | Y | Y |  |  |  |  |  |
| Bavare et al., 2018 | **4** | Y | Y |  |  |  |  |  | Y | N | Y | Y | Y |  |  |  |  |  |
| Bogert et al., 2010 | **3** | Y | Y |  |  |  |  |  | Not clear | Y | Y | Not clear | Y |  |  |  |  |  |
| Brady et al., 2015 | **5** | Y | Y |  |  |  |  |  | Y | Y | Y | Y | Y |  |  |  |  |  |
| Carter et al., 2022 | **5** | Y | Y | Y | Y | Y | Y | Y | Y | Y | Y | Y | Y | Y | Y | Y | Y | Y |
| Cornell and Datson, 2023 | **3** | Y | Y |  |  |  |  |  |  |  |  |  |  | Y | Y | Not clear | Y | Not clear |
| Dwyer et al., 2020 | **5** | Y | Y |  |  |  |  |  | Y | Y | Y | Y | Y |  |  |  |  |  |
| Eden et al., 2017 | **5** | Y | Y |  |  |  |  |  | Y | Y | Y | Y | Y |  |  |  |  |  |
| Flynn et al., 2022 | **5** | Y | Y |  |  |  |  |  | Y | Y | Y | Y | Y |  |  |  |  |  |
| Gerdik et al., 2010 | **4** | Y | Y |  |  |  |  |  | Y | Y | Y | N | Y |  |  |  |  |  |
| Gill et al., 2022 | **2** | Y | Y | Y | Y | Not clear | N | Not clear |  |  |  |  |  |  |  |  |  |  |
| Gill et al., 2021 | **5** | Y | Y | Y | Y | Y | Y | Y |  |  |  |  |  |  |  |  |  |  |
| Gill et al., 2018 | **5** | Y | Y | Y | Y | Y | Y | Y |  |  |  |  |  |  |  |  |  |  |
| Gill et al., 2019 | **4** | Y | Y | Y | Y | Y | Y | Y | Y | Y | Y | Y | Y | Y | Y | Y | N | Y |
| Guinane et al., 2018 | **5** | Y | Y | Y | Y | Y | Y | Y |  |  |  |  |  |  |  |  |  |  |
| Heath et al., 2016 | **5** | Y | Y | Y | Y | Y | Y | Y |  |  |  |  |  |  |  |  |  |  |
| Hueckel et al., 2012 | **1** | Y | Y |  |  |  |  |  | N | N | N | N | Y |  |  |  |  |  |
| King et al., 2019 | **5** | Y | Y | Y | Y | Y | Y | Y |  |  |  |  |  |  |  |  |  |  |
| King et al., 2024 | **5** | Y | Y | Y | Y | Y | Y | Y |  |  |  |  |  |  |  |  |  |  |
| Marufu et al., 2025 | **5** | Y | Y | Y | Y | Y | Y | Y |  |  |  |  |  |  |  |  |  |  |
| McCawley et al., 2013 | **5** | Y | Y |  |  |  |  |  | Y | Y | Y | Y | Y |  |  |  |  |  |
| New et al., 2019 | **5** | Y | Y | Y | Y | Y | Y | Y |  |  |  |  |  |  |  |  |  |  |
| Ngoya Ntumba et al., 2023 | **5** | Y | Y | Y | Y | Y | Y | Y |  |  |  |  |  |  |  |  |  |  |
| Odell et al., 2010 | **2** | Y | Y | Y | N | Y | Not clear | N |  |  |  |  |  |  |  |  |  |  |
| O’Halloran et al., 2023 | **4** | Y | Y | Y | Y | Not clear | Not clear | Not clear | Y | Y | Y | Y | Y | Y | Y | Y | Y | N |
| Strickland et al., 2019 | **5** | Y | Y | Y | Y | Y | Y | Y |  |  |  |  |  |  |  |  |  |  |
| Sutton et al., 2024 | **5** | Y | Y | Y | Y | Y | Y | Y |  |  |  |  |  |  |  |  |  |  |
| Thiele et al., 2022 | **5** | Y | Y | Y | Y | Y | Y | Y |  |  |  |  |  |  |  |  |  |  |
| Thiele et al.,2025 | **5** | Y | Y | Y | Y | Y | Y | Y |  |  |  |  |  |  |  |  |  |  |
| Earle et al., *2018* | **5** | Y | Y | Y | Y | Y | Y | Y |  |  |  |  |  |  |  |  |  |  |
| Price et al., *2028* | **5** | Y | Y | Y | Y | Y | Y | Y |  |  |  |  |  |  |  |  |  |  |
| Odell, 2019 | **4** | Y | Y |  |  |  |  |  | Y | Y | Y | Not clear | Y |  |  |  |  |  |
| Van Voorhis and Willis, 2009 | **2** | Y | Y |  |  |  |  |  | Y | Y | Not clear | Not clear | Not clear |  |  |  |  |  |
| See et al., 2014 | **4** | Y | Y |  |  |  |  |  | Y | N | Y | Y | Y |  |  |  |  |  |

No included papers were quantitative randomized or non-randomized controlled trials. These categories were withdrawn from the above table for lisibility purposes.

**Mixed Methods Appraisal Tool (MMAT, version 2018) questions:**

| **Category of study** | **Methodological quality criteria** |
| --- | --- |
| Screening questions  (for all types) | S1. Are there clear research questions?  S2. Do the collected data allow to address the research questions? |
| 1. Qualitative | 1.1. Is the qualitative approach appropriate to answer the research question?  1.2. Are the qualitative data collection methods adequate to address the research question?  1.3. Are the findings adequately derived from the data?  1.4. Is the interpretation of results sufficiently substantiated by data?  1.5. Is there coherence between qualitative data sources, collection, analysis and interpretation? |
| 2. Quantitative randomized controlled  trials | 2.1. Is randomization appropriately performed?  2.2. Are the groups comparable at baseline?  2.3. Are there complete outcome data?  2.4. Are outcome assessors blinded to the intervention provided?  2.5 Did the participants adhere to the assigned intervention? |
| 3. Quantitative non-  randomized | 3.1. Are the participants representative of the target population?  3.2. Are measurements appropriate regarding both the outcome and intervention (or exposure)?  3.3. Are there complete outcome data?  3.4. Are the confounders accounted for in the design and analysis?  3.5. During the study period, is the intervention administered (or exposure occurred) as intended? |
| 4. Quantitative  Descriptive | 4.1. Is the sampling strategy relevant to address the research question?  4.2. Is the sample representative of the target population?  4.3. Are the measurements appropriate?  4.4. Is the risk of nonresponse bias low?  4.5. Is the statistical analysis appropriate to answer the research question? |
| 5. Mixed methods Supplemental material | 5.1. Is there an adequate rationale for using a mixed methods design to address the research question?  5.2. Are the different components of the study effectively integrated to answer the research question?  5.3. Are the outputs of the integration of qualitative and quantitative components adequately interpreted?  5.4. Are divergences and inconsistencies between quantitative and qualitative results adequately addressed?  5.5. Do the different components of the study adhere to the quality criteria of each tradition of the methods involved? |

## Appendix 4: Additional tables

**Table 2: Existing PFAES identified in the included studies (full table).**

| **Characteristics** | | | | | **Core design features** | | | | | **Population and setting** | | |  |
| --- | --- | --- | --- | --- | --- | --- | --- | --- | --- | --- | --- | --- | --- |
| **Name** | **Country (Region / State if stated)** | **Year of implementation (if stated)** | **Health system level** | **Implementation stage** | **Who can activate escalation** | **PFAES activation channel** | **Target responder** | **Scope** | **Gatekeeping** | **Hospital type** | **Service** | **Target population** | **Ref** |
| 4-CARE | United States, Florida | 2007 | Local | Post- implementation (2 years) | Patients (adult); Families | Phone | RRT | Deterioration | Direct | Tertiary teaching hospital | All | Adult | [18] |
| AWARE | Singapore | 2012 | Local | Pilot | HCP | In-person | HCP | Deterioration | Primary healthcare team | Acute tertiary hospital | Two general medical-surgical wards | Adult | [51] |
| Call 4 Concern | United Kingdom, England | 2009 | Local | Pilot & post implementation (7 years) | Patients (adult); Families | Phone | CCOT | Escalation of concerns | Direct | District General Hospitals | All | Adult & Paediatrics | [24,30,49] |
| Calling 4 Help (C4H) | Australia, Western Australia | 2015 | Local | Implementation | Families | Phone | MET | Deterioration | Direct | Tertiary paediatric hospital | All | Paediatrics | [35,36] |
| Consumer Escalation System (CES) | Australia,  South Australia | 2019 | Local (from national requirements) | Implementation | HCP | In-person | RRT | Escalation of concerns | Primary healthcare team | Public teaching hospital | Medical, surgical, and speciality wards | Adult | [44,45] |
| Condition Concern | United States, Michigan | 2009 | Local | Implementation | Patients; families | Phone | Administrative Associate Manager (forwards to RRT) | Escalation of concerns | Triage | Three not-for-profit major hospitals | Not reported | Adult & Paediatrics | [20] |
| Condition H | United States, California | 2007 | Local | Pilot | Patients (adult); Families; Visitors | Phone | RRT | Deterioration | Direct | Tertiary hospitals | All | Adult | [22] |
|  | United States, North Carolina | 2007 |  |  |  |  |  |  |  |  |  |  | [29] |
| Condition H(elp) | United States, North Carolina | 2011 | Local | Post-implementation (1 year) | Families | Phone | RRT | Escalation of concerns | Direct | Tertiary paediatric hospital | Two inpatient paediatric units | Paediatrics | [27] |
| Condition Help (CH) | United States, Pennsylvania | 2005 | Local | Post implementation (3 years) | Patients (adult); Families | Phone | RRT | Escalation of concerns | Direct | Tertiary teaching hospital | All | Adult & Paediatrics | [32] |
| Consumer-initiated escalation-of-care (CIEoC) | Australia, South Australia | 2019 | Local | Post-implementation (3 years) | Patients; Families; Visitors | Phone | RRT / MET | Deterioration | Direct | Two hospitals (Trauma medical centre; Acute Care Hospital) | All | Adult & Paediatrics | [28] |
| Detect E-PEWS | United Kingdom, England | 2020 | Local | Implementation | HCP | Digital (app) | Ward clinical team & RRT | Deterioration | Primary healthcare team | Tertiary paediatric hospital | All | Paediatrics | [23] |
| Early 3S’ (RESPOND) | United Kingdom, England | 2022 | Local | Implementation | Patients (adult) | Digital (buzzer) | CCOT / MET | Escalation of concerns | Direct | Teaching hospital, rural DGH, urban DGH | Selected wards (4 total) | Adult | [33] |
| ESCALATION | Australia, Western Australia | 2021 | Regional | Implementation | HCP | In-person | Case-by-case, based on EWS | Deterioration | Primary healthcare team | Tertiary paediatric hospital | All | Paediatrics | [25,47] |
| Family Activated MET | United States, Ohio | 2007 | Local | Implementation; Post-implementation (6 years) | Families | Phone | MET | Escalation of concerns | Direct | Tertiary paediatric hospital | All non-ICU in-patient units | Paediatrics | [40] |
| Family-Initiated Rapid Response (FIRR) | United States, Texas | 2009 | Local | Post-implementation (3 years) | Families | Phone | RRT | Escalation of concerns | Direct | Tertiary paediatric hospital | Non-critical care inpatient units | Paediatrics | [21] |
| Korero Mai | New Zealand, Canterbury | 2018 | National | Implementation | HCP | In-person | Primary HCP | Escalation of concerns | Primary healthcare team | Tertiary regional hospital | Paediatric progressive care/high dependency unit | Paediatrics | [41] |
|  | New Zealand, Waitemata | 2018 |  |  | Patients; Family; Whānau | Phone & in-person | Independent clinical reviewer & Primary HCP |  | Direct & Primary healthcare team | District Health Board | Not reported | Adult & Paediatrics | [42] |
| Listening to You | United Kingdom, England | 2014 | Local | Implementation | HCP | In-person | Ward clinical team & PACE | Deterioration, communication bundle | Primary healthcare team | Tertiary paediatric hospital | Four inpatient wards | Paediatrics | [26] |
| PARR | United Kingdom, Wales | 2023 | Local | Implementation | Patients (adult); Families | Phone | RRT | Escalation of concerns | Direct | District General Hospital | Single ward and discharged patients | Adult | [38] |
| Pediatric Care BefOre Deterioration Events (CODE) | United States | Not reported (data collection started 2021) | National | Not reported | HCP | In-person | Primary HCP | Escalation of concerns | Primary healthcare team | 30 medical centres (including 21 paediatric hospitals) | Paediatrics | Paediatrics | [17] |
| Pediatric rapid response system (PRRS) | United States, North Carolina | 2005 | Local | Implementation | Families | Phone | RRT | Deterioration | Direct | Tertiary paediatric hospital | All | Paediatrics | [50] |
| Ryan’s Rule | Australia, Queensland | 2013 | Regional | Post-implementation (5 years) | Patients; Families; Friends | Phone | government hotline > Hospital contact (forwards to clinical review) | Deterioration | Triage | Ten hospitals | Surgical ward | Adult & Paediatrics | [39] |
|  |  |  |  | Post-implementation (2 years) | Patients; families | Phone | Independent clinical reviewer |  | Direct | Tertiary regional hospital | All |  | [46] |
| Stop-alert; Assess-fix-eliminate; Escalate and report | Canada, Saskatoon | Not reported (data collection started 2017) | Local | Post-implementation (missing year of implementation) | Patients (adult); Families | Phone | Primary HCP | Escalation of concerns | Direct | Urban tertiary care centre | One surgical and three medical inpatient wards | Adult | [43] |

**Table 6a**: Themes related to HCP perception and responses to PFAES in included study.

|  | | [14] | [15] | [17] | [18] | [19] | [21] | [22] | [26] | [28] | [29] | [31] | [32] | [34] | [35] | [36] | [39] | [42] | [45] | [46] | [48] |
| --- | --- | --- | --- | --- | --- | --- | --- | --- | --- | --- | --- | --- | --- | --- | --- | --- | --- | --- | --- | --- | --- |
| Population | HCP (ward staff) | x | x | x | x | x | x | x | x | x | x | x | x | x | x | x | x | x | x | x | x |
|  | HCP (managers) |  | x | x |  |  |  |  | x |  |  |  |  |  |  |  | x | x | x |  |  |
| Expectations  (pre-implementation) | Workload increase |  | x |  | x |  | x |  |  | x | x |  |  | x | x | x |  |  |  | x | x |
|  | Non-urgent use |  | x |  | x |  | x |  |  | x | x |  |  | x | x | x |  |  |  | x | x |
|  | Resources constraints |  | x |  | x |  | x |  |  |  |  |  |  | x | x |  |  |  |  | x | x |
| Authority and relational dynamics | Threat to professional judgement |  |  |  |  | x |  |  |  |  |  |  |  |  |  |  |  |  |  | x |  |
|  | Relations strain between HCP and patient/family |  |  |  |  |  | x |  |  | x |  | x |  |  | x | x |  |  | x | x |  |
|  | Fear of professional repercussions |  |  |  |  |  |  |  |  | x |  |  |  |  |  |  |  |  |  | x |  |
| Bypass and capability | Bypass opposition |  |  |  |  |  |  |  |  |  |  |  |  |  |  |  |  |  |  | x |  |
|  | Doubt family judgement | x |  |  |  |  |  |  |  | x |  | x |  |  |  |  |  |  | x | x |  |
| Sustained delivery | Ongoing scepticism |  |  | x |  |  |  |  |  |  |  |  |  |  |  |  |  |  | x | x |  |
| Gatekeeping | Selective informing of patients and families |  |  | x |  |  |  |  |  | x | x |  |  |  |  |  |  |  |  | x |  |
|  | Concerns as complaints |  |  |  |  |  | x |  |  |  |  |  |  |  |  |  |  |  |  |  |  |
| Positive value | Family-centred fit |  |  |  |  |  |  | x |  | x | x |  |  |  |  |  |  | x |  |  |  |
|  | Perceived safety gains |  |  |  |  | x |  |  |  |  |  |  |  | x |  |  |  |  | x | x |  |

**Table 6b**: Themes related to patient and family perception and responses to PFAES in included study.

|  |  | [14] | [15] | [16] | [17] | [18] | [19] | [20] | [21] | [22] | [23] | [24] | [25] | [26] | [27] | [28] | [29] | [30] | [31] | [32] | [35] | [36] | [40] | [41] | [43] | [44] | [45] |
| --- | --- | --- | --- | --- | --- | --- | --- | --- | --- | --- | --- | --- | --- | --- | --- | --- | --- | --- | --- | --- | --- | --- | --- | --- | --- | --- | --- |
| Population | Patients | x | x | x | x | x |  | x | x |  | x | x | x | x |  | x |  | x | x |  |  | x | x | x | x | x | x |
|  | Family members and carers |  | x | x | x | x | x |  | x | x |  |  | x | x |  | x | x |  | x | x | x |  | x |  |  |  |  |
| Perception | Increases patient safety |  |  |  |  |  | x |  |  |  |  |  |  |  |  |  |  |  |  |  | x |  |  |  |  | x |  |
|  | Supportive messaging |  |  |  |  |  |  |  |  |  |  |  |  |  |  |  |  | x |  |  |  |  |  |  |  | x |  |
| Barriers to speaking up | Perceived lack of capability |  | x |  |  |  | x |  |  | x |  |  |  |  | x | x | x | x | x |  | x |  |  | x | x | x |  |
|  | HCP relationship concerns |  |  |  |  |  |  |  |  |  |  |  |  |  | x |  |  |  | x |  |  |  | x | x |  | x |  |
|  | Seeming impolite |  |  |  |  |  |  |  | x |  |  |  |  |  |  | x | x | x |  |  |  |  |  |  |  | x |  |
|  | Fear of reprisals |  | x |  |  |  |  |  |  |  |  |  |  |  |  |  |  |  | x |  |  |  |  |  |  |  |  |
|  | Reluctance to burden staff |  |  |  |  |  |  |  |  | x |  |  |  |  |  |  | x | x |  |  | x |  |  |  |  | x | x |
|  | Doubts about staff response | x |  |  |  |  |  |  |  | x |  |  |  | x |  |  |  |  | x |  |  |  |  |  |  |  |  |
| Preference for proxy escalation | Deference to hierarchy |  |  |  |  |  |  |  |  |  |  |  |  |  |  |  | x |  |  |  |  |  |  |  |  | x |  |
|  | Trust in HCP |  |  |  |  |  |  |  |  |  |  |  |  |  |  |  |  |  |  | x |  |  |  |  |  | x |  |
|  | Perceived role boundaries |  |  |  |  |  |  |  |  |  |  |  |  |  | x |  |  | x |  |  |  |  |  |  |  |  |  |
| Conditions for self-efficacy | Self-confidence in recognising deterioration |  |  |  |  |  |  |  |  |  |  |  |  |  |  | x |  |  |  |  | x |  |  |  |  |  |  |
|  | Staff-enabled confidence |  |  |  |  |  |  |  |  | x |  |  |  |  |  |  |  |  |  |  |  |  |  |  |  |  |  |
|  | Clear communication |  |  |  | x |  |  |  |  |  |  |  |  |  | x |  |  |  |  |  |  |  |  |  |  |  |  |

**Table 7**: Themes related to PFAES effectiveness and sustainability by included study

|  |  | [17] | [18] | [20] | [21] | [22] | [23] | [24] | [25] | [26] | [27] | [28] | [29] | [30] | [32] | [33] | [35] | [36] | [37] | [38] | [39] | [40] | [41] | [42] | [43] | [44] | [45] | [46] | [47] | [49] | [50] | [51] |
| --- | --- | --- | --- | --- | --- | --- | --- | --- | --- | --- | --- | --- | --- | --- | --- | --- | --- | --- | --- | --- | --- | --- | --- | --- | --- | --- | --- | --- | --- | --- | --- | --- |
| Implementation stage | <12 months | x |  | x |  | x | x |  | x | x |  |  | x |  |  | x | x | x | x | x |  |  | x | x | x | x | x |  | x |  | x | x |
|  | ≥12 months |  | x |  | x |  |  | x |  |  | x | x |  | x | x |  |  |  |  |  | x | x |  |  |  |  |  | x |  | x |  |  |
| Barriers to sustainability | Limited leadership |  |  |  |  |  |  | x |  |  |  |  |  |  |  | x | x |  |  |  |  |  |  |  |  |  |  |  |  |  |  |  |
|  | Lack of role clarity |  |  |  |  |  |  |  |  | x |  |  |  |  |  |  | x |  |  |  |  |  | x |  |  | x |  |  |  |  |  |  |
| Enabler to sustainability | Governance and leadership | x |  |  |  | x | x | x |  |  | x | x | x |  |  | x | x |  |  |  |  | x | x | x |  |  | x | x | x | x | x |  |
|  | Operational mechanisms |  |  |  |  | x | x |  |  |  | x | x | x |  |  | x |  |  |  |  |  |  | x |  |  |  |  | x |  |  |  |  |
|  | Routine integrations |  |  |  |  |  |  | x |  |  |  |  |  |  | x |  |  |  |  |  |  | x | x | x |  |  | x | x |  |  |  |  |
|  | Staff support and tools |  | x |  |  |  |  |  |  |  |  |  |  |  |  |  |  |  |  |  |  |  |  |  |  |  |  |  | x |  |  |  |
|  | Ongoing promotion |  | x |  | x | x |  | x |  | x |  |  | x |  |  |  |  |  | x |  |  |  | x | x |  |  | x |  | x | x |  |  |
| Barriers to effectiveness | Response delays |  |  |  |  |  |  |  |  |  |  |  |  |  | x |  |  |  |  |  | x |  |  |  |  |  |  | x |  |  |  |  |
|  | Interprofessional communication |  |  |  |  |  |  |  |  |  |  |  |  |  |  |  |  | x |  |  |  |  | x |  |  |  |  |  | x |  |  |  |
|  | Hierarchies and boundaries |  |  |  |  |  |  |  |  | x |  |  |  |  |  |  |  |  |  |  |  |  |  |  |  |  |  |  | x |  |  |  |
| Enabler to effectiveness | Organisational resourcing |  |  |  |  |  |  |  |  |  |  | x |  | x |  |  |  |  |  |  |  |  |  |  |  |  |  |  |  |  |  |  |
|  | Leadership buy-in |  |  |  |  |  |  |  |  |  |  |  |  |  |  |  |  |  |  |  |  |  |  |  |  |  |  |  |  |  |  |  |

**Table 8**: Themes related to PFAES effectiveness and sustainability by included study

|  |  | [19] | [21] | [22] | [23] | [24] | [25] | [26] | [27] | [28] | [29] | [31] | [32] | [33] | [34] | [35] | [36] | [37] | [39] | [41] | [42] | [43] | [45] | [48] | [49] | [50] | [51] |
| --- | --- | --- | --- | --- | --- | --- | --- | --- | --- | --- | --- | --- | --- | --- | --- | --- | --- | --- | --- | --- | --- | --- | --- | --- | --- | --- | --- |
| Equity reporting | Diverse sample |  |  |  |  |  | x |  |  |  |  |  | x |  | x |  |  |  |  | x | x | x |  | x |  |  |  |
|  | Acknowledgement of limited equity data | x |  |  | x |  |  |  |  |  |  |  |  |  |  |  |  |  |  |  |  |  |  |  |  |  |  |
|  | Exclusion of non-English speaking participants |  |  |  |  |  |  |  |  |  |  | x |  |  |  |  |  |  |  |  |  |  | x |  |  |  | x |
|  | Limited demographic data |  | x | x |  | x |  | x | x |  |  |  |  |  |  | x | x |  |  |  |  |  |  |  | x |  |  |
| Access barriers | Language |  |  | x |  |  | x |  |  | x |  |  |  | x |  | x | x |  | x |  | x |  | x |  |  | x |  |
|  | Interpreter provision |  |  |  |  |  | x |  |  |  | x |  |  |  |  |  |  |  |  |  |  |  |  |  |  |  |  |
|  | Inequities in HCP engagement |  |  |  |  |  | x | x |  |  | x |  |  | x |  |  | x |  |  |  |  |  | x |  |  |  |  |
|  | Sociocultural norms against questioning clinicians |  |  |  |  |  | x |  |  |  |  |  |  | x |  |  |  | x |  |  | x |  | x | x |  |  |  |
| Access enablers | Adapted resources |  |  |  |  |  | x |  |  | x | x |  |  |  | x |  | x | x |  |  | x |  |  |  |  | x |  |
|  | Adequate services |  |  |  |  |  | x |  |  | x |  |  |  |  |  |  |  |  |  |  |  |  |  |  |  |  |  |
|  | Legitimising escalation |  |  |  |  |  |  |  |  |  |  |  |  | x |  |  |  | x |  |  | x |  |  |  |  |  |  |
|  | Inclusive implementation |  |  |  |  |  | x |  |  |  |  |  |  |  |  |  |  |  |  | x | x |  |  |  |  |  |  |
|  | Practical accessibility adaptations |  |  |  |  |  |  |  |  |  | x |  |  |  | x |  |  |  |  |  |  |  |  |  |  | x |  |
